# A Few Notes on the Morbid Anatomy and Pathology of Varix and Allied Affections, Especially as They Occur in the Veins of the Lower Extremities

**Published:** 1897-04

**Authors:** Thomas H. Manley

**Affiliations:** Professor of Surgery at the New York School of Clinical Medicine; New York


### A FEW NOTES ON THE MORBID ANATOMY AND PATHOLOGY OF VARIX AND ALLIED AFFECTIONS, ESPECIALLY AS THEY OCCUR IN THE VEINS OF THE LOWER EXTREMITIES.

By THOMAS H. MANLEY, M. D.,
Professor of Surgery at the New York School of Clinical Medicine.

There is no infirmity of the human body more common or more distressing than that which, in various regions, succeeds in consequence of varix of various plexuses of veins, notably those of the vena-saphena, saphena-interna, the vena-cruralis-profunda, and the gluteal.

Our knowledge of the anatomical elements of the veins, their mode of development, their deviation in growth, with the clear and irresistible influence of heredity, all incline us to the view that the fundamental element in their evolution is congenital defect, probably in the muscular wall of the vessel.

A proper understanding of exactly what the pathological factors of confirmed varix are, will enable us to determine what we may hope to attain by treatment in the simple or the complicated cases.

It is a matter of common observation that there are many defects of development and hereditary tendencies which we only begin to become conscious of after we reach adult years, or approach middle life.

In this class, *par excellence*, are varicose veins, the full evolution of which, however, depends in a large measure on environment, physiological state, or occupation, etc.

It does not appear that the pathology of varicose veins was ever seriously studied until early in the present century.

Crisp tells us that inflammation in the veins was first described in England by John Hunter in 1793. (*Diseases of the Blood Vessels.*) "It appears," he says, "principally in two forms; in one, there is an accumulation of pus in the vessel, and in the other the lumen is stuffed by fibrous coagula. The parturient condition, and constitutional states of the blood seem to favor it."

Trauma, the incision in phlebotomy, or contusion, was said to favor it. Guthrie declared that in the Peninsular War hundreds of the wounded lost their lives through it. In his experience it was the special dread of the surgeon after amputations. (*Cooper's Surg. Dict., p. 211.*)

Cruveilher believed that it occasioned a greater number of deaths after all operations in Paris than any other cause. (*Dict. de Med. et de Chi., Vol.II., p. 412.*)

Brodie described the symptoms which ushered in phlebitis as similar to those of typhoid. (*Diseases of the Veins, Brodie, p. 211.*)

Robin has published an account of the rupture of a varicose vena-cava-descendens in a young man, during a sparring match.

The vessel gave way over the second lumbar vertebra. Its wall was very thin and there was practically an absence of the muscular coat.

This author divides rupture of veins into three classes: First, the anatomical; second, traumatic; third, those caused by ulceration in adjoining structures. (*Jour. de Med., Bordeaux, December, 1894.*)

Cumbly, in January, 1894, presented a patient to the French Academy who had complete obliteration of the superior vena-cava. Patient was confined to bed two years after the vessel closed, after which time the surface collaterals became enormously distended; the principal vessels over the thorax becoming as large as the femoral.

The author believed that occlusion came in consequence of tremor pressure in the anterior mediastinal space. (*Bull. de l'Acad. de Med., Feb., 1894.*)

Delageniere notes the influence of deep varix as a factor in sciatica. (*Sciatica-femero-fessiere, de l'origin variqueuse. Traitee avec succes par le denudation et de la dissection du nerf.—Revue de Chirurg., 10 Juillet, 1896.*)

His patient had a most rebellious type of sciatica, which resisted the usual remedies and nerve-stretching.

The surgeon, remembering Quenu's views on varix as a causative factor in sciatica, proceeded to denude the nerve, when, to his astonishment, he found that not only was the nerve tightly compressed by large, thickened leashes of varicose veins, but some of them penetrated its sheath.

The diseased veinous plexus was dissected away, the vessels ligated, and the nerve trunks completely liberated. Recovery was prompt and permanent.

Dr. John Hunter of Toronto, reports a case of aphasia and death following thrombosis of the femoral vein. (*Canadian Practitioner, Nov. 16, 1891.*)

### VARICOSE VEINS OF THE LOWER EXTREMITY.

Although we may find varix in any part of the body, where there are veins, except in the osseous structures, their most common situation, as well as where they may be studied with the most advantage, is in the lower extremity.

It is in this situation we can observe, too, the many complications which attend this evolution, and sequelæ resulting.

It has long been observed that the veins are more prone to disease than the arteries, as we can easily understand when we consider that they are much less specialized structures, they lie more superficially, and they convey vitiated, deoxygenated blood, which moves through them in a languid current.

There are two large sets of veins provided for the extremities, the deep and the superficial, both freely communicating with each other; the deeper, more constricted draining into the more capacious surface or sub-aponeurotic by narrow inter-connecting loops, provided with valves which permit the blood to only move in an outward direction. According to Reclus, the deep veins are always the first to show signs of disease, the peripheral becoming involved later. (*Duploz et Reclus*, *Chirurg*, *Vol. II*, *p. 209.*) But Valettte, and later Hughes (*British Med. Jour.*, *May 12*, *1887.*), take exception to this view; the latter citing a case of extensive varix of the saphena, in which the deep veins were all found healthy.

Disease of the veins of the extremity is made manifest by various distinct pathological changes. These may be divided into—

First, *Stasis*, *turgescence*, or *congestive.*

Second, *Inflammation*, *periphlebitis*, *endophlebitis.*

Third, *Thrombosis*, *obliterative atresia*, *phlebectasia*, or *vascular rupture with hemorrhage.*

Complications and consecutive changes in the subjacent structures.

First, *Dermatitis*, *eczema*, and *pigmentation.*

Second, *Ulceration*, *pruritus*, *and degenerative wasting of all the structures of the limb.*

Third, In rare cases with acute obliterative inflammation and localized gangrene.

Many afflicted with varix, in its early stages, are conscious of a sense of fatigue, numbness, or pain over various areas of the lower extremity, especially in the thigh or leg, when they stand long or walk long distances. They notice on retiring at night that the affected limb is more or less œdematous near the ankle joint. If the individual is inclined to be fat, the distended superficial veins are not visible, but on deep pressure their tortuous, cordy character is readily detected.

There are many who go through life with practically no further advance of this infirmity, aggravated by gravity or overstrain; those who otherwise enjoy good health, who live regular lives, and are not compelled to perform severe physical labor; who are free from a marked rheumatic diathesis, and have never had syphilis. But with the working man or woman it is hard to conceive of an affliction, not of a malignant character, productive of greater misery; for with them the infirmity almost invariably advances into the second stage of

### INFLAMMATION.

And who can calculate the number of very grave pathological conditions in distant organs, or of sudden deaths, which varicose veins of the limbs are indirectly responsible for?

It requires no stretch of the imagination to conceive of very many serious consequences succeeding the displacement of a a thrombus, sent into the general circulation and swept by the blood on through the lungs to plug the ramifications of the pulmonary artery and induce no end of mischief, when the asphyxiated area becomes infected from the bronchial tubes.

Considering the great frequency of varix and its remote, but no doubt great, influence as a factor in pulmonary diseases, it may, perhaps, often be well, before we decide on the probable etiological factors of a given case of lung disease, in one past middle life, to first inspect the state of the veins of the extremities.

How often those with varix suffer from cramp in the calf —stagnation of the blood—and is it not probable that the "stitch in the side," possibly, the initial stitch of pleurisy, is primarily attributable to a thrombus carried from the stuffed saphena into the pulmonary tide.

Therapeutic facts point strongly that way. For example, any practitioner properly trained in the therapeutics of pleurisy, knows that in its initial stages free venous depletion is its sovereign remedy, will arrest its march at the first stage, by a remedy which is not empirical, but strikes at the root of the trouble, in relieving the localized asphyxia of the crippled organ.

Last autumn, it was my privilege to submit a brief brochure before the New York State Association, which dealt with the etiology of some of the neuralgias, in which the ground was taken that the blood itself is a positive medium for the transmission of sensory impulses (*Vascular Impediment and Localized Asphyxia as an Etiological Factor in the Production of Pain—.Transactions of New York State Medical Association, 1896*). This, it has been in my power to repeatedly demonstrate, both experimentally and clinically, in the animal and man.

From this short digression let us return to the local changes. In inflammation of the veins the nerves are starved, and the action of the glands perverted. The rete malpigia becomes first, thickened, then hard; later, the horny layer dries and fissures, the over-stimulated sudoriparous glands pouring their briney secretions into the numerous nude interstices. The skin in places becomes bronzed, thinned and glossy. The vessels are felt enlarged and lumpy; their walls are thickened. In various places we will find segments of the vessel plugged with coagula, or distended with black, tarry blood.

In some instances, where I have dissected out the saphena-interna, large, shell-like incrustations of the lime salts have been found, not within the lumen of the vessel, but between the interna and the muscalaris.

In other words we will find venous wells in places where the vessel has become sacculated and widely distended.

These are most commonly found in the popliteal space, over Scarpa's triangle, and in the veins of the labium-majus of the vulva in women.

The valves are generally found, ruptured, sclerosed, or resorbed. They are, in all aggravated cases, quite functionless; and hence the reason of danger to life, from mortal hemorrhage, when a large varicose trunk opens externally, as the blood flows from above as well as below. In order to provide for the support to the column of blood against gravity, we must have a compensatory hypertrophy of vascular walls.

As the disease advances, one set of veins after another becomes involved. A low grade of inflammation seizes on the walls of the vessels and extends in various directions, finally involving all the structures. The integument, however, suffers to the greatest extent; as its glandular elements are destroyed, its nerves simply impoverished, and its integrity radically undermined, its vitality and resistance are greatly enfeebled, so that in the event of irritation or contusion of it, it readily takes on inflammation, breaks down, or takes on degenerative changes, when we have that distressing lesion, known as varicose-ulcer.

### ULCERATION IN VARIX.

There are many features of varicose ulcer that bear a close analogy to that of cancer. When one of these lesions is well under way, we have an enormous weeping of the epithelia. This discharge is serous, dark-colored and foul-smelling. The edges of the ulcer are calloused and insensitive, and differ from the indurated border of an epithelioma, in that they are not so prone to bleeding on slight irritation.

We have a large variety of varicose ulcer of the leg, too numerous to deal with separately here. It is quite enough to say that they depend on both local and general conditions. My experience has long since convinced me, that various constitutional disturbances, and organic maladies play an important role in the pathology of ulcerations of a benign order nor am I above the charge of sufficient of old fogyism to entirely discard the views of some of our older authors, who regarded the moderate discharge from an hemorrhoidal ulcer —commonly known as fistula and always primarily dependent on rectal varix—and others, as sometimes, under certain disordered states of the system, as not without certain salutary purpose.

There can be no qestion, but scurvy, rheumatism, syphilis, tuberculosis and other systemic diseases, bear something more than a casual relation to progressive, benign ulceration.

This description of ulcer is always more troublesome in the warm season of summer, when the system is enervated by the depressing effect of great heat. The distress which they occasion in the heated spell is something extremely trying on those in life who are compelled to perform heavy manual labor for a living.

Happily, nature frequently comes to their relief, for of all severe bodily infirmities, there is none better borne than varicose ulcer. In some we will notice that although we discover a wide breach in the continuity of the tissues, the individual is quite insensitive to pain; nor is there any tissue more docile to well directed remedial measures.

In many of these cases nature provides a cure, or a relief of the more urgent symptoms by changes in or about the walls of the veins.

### PHLEBITIS—OBLITERANS AND THROMBOSIS.

Oftentimes, relief comes, through obliterative phlebitis; we have endothelial proliferation and whole territories shut out by inflammatory atresia; and again, the same result ensues from multiple thromboses of the peripheral districts; the imprisoned blood undergoing organization, transposing the vessels into a whip-cord consistence.

We occasionally meet with instances in which this transformation in the vascular walls sets in insensibly. Several such cases have come to my notice here in New York, among rejected candidates of the police and fire departments. According to the statements of these individuals, their rejection because of the "broken veins" was the first intimation that they ever had them.

Localized gangrene as a result of varix has been denied; but in a large, fat, diabetic old woman, who was under my care, there was nothing else to account for it. There was no evidence of arterial impediment, as the pulsation of the dorsalis-pedis and posterior-tibial was distinctly felt. It was interesting to observe in this case that although the extent of sphacelus was considerable and the inner surface of part of the tibial shaft exposed, yet repair was rapid and complete.

### CONSTITUTIONAL CONDITIONS AS DETERMINING FACTORS OF LOCAL VARIX.

Modern investigators have repeatedly demonstrated that multiple phlebitis is an invariable concomitant of acute inflammatory rheumatism. The poison of this infirmity is well known to seize on, by selection, the fibro-serous structures, those invested by an endothelial lining. The probabilities are that in rheumatic endocarditis, pneumonia and pleurisy, the thoracic organs are involved secondarily by detached infected emboli, carried in the circulation from distant areas of diseased veins. Neither can there be little doubt, but, in a considerable proportion of those localized and painful areas, set down as "neuralgic or rheumatic," the real seat of pathologic change is in the veins; there is a phlebectasia, a phlebitis or a thrombosis, and great distension of the more deeply concealed veins.

Pain and functional impediment follow and persist, until the clot breaks down, or an ample collateral circulation is established; something readily effected with the venous system, which is so much more abundantly provided with anastomosing communicating branches than the arterial.

### PHLEGMASIA—ALBA DOLENS.

So-called milk-leg, a condition nearly always in some manner connected with the parturient state, falls with great force on the veins.

The pathology of this malady is certainly yet imperfectly understood, although with its baneful effects all are acquainted. Varquez believes that it is but a second stage of a complication of infection. Socrelle, in autopsies in 233 cases of death from puerperal fever, found inflammation of the veins and lymphatics in 110. Dr. Robert Lee believed that in these cases the inflammation commenced in the radicles of the hypogastric veins; from thence propagated to the iliac, femoral and saphenæ. He believed that the uterine sinuses remained open, and that after delivery a condition similar to amputation followed.

White, Tyre, and others have endeavored to prove that these primary pathologic changes are in the lymphatic channels. Muscagni and Panozzi, in the latter part of the last century, were first led on to the discovery of the lymphatic system while studying the morbid anatomy of phlegmasia-dolens. (*Descriptions et Tenographie des Vaisseaux Lymphatics.—Sappey, Vol. 3, p. 270.*) All investigators agree that in this obscure disease the lymphatic system is extensively involved.

But the most palpable and extensive changes are found in the peripheral veins of the greatest calibre. In some, the healthy vein is firmly closed by dense clots; in others the vessel undergoes sufficient thickening to close its lumen; while in others yet, the thrombus parts with its hæmatine, becomes disentegrated, or organized and fixed.

In aggravated cases all the soft parts share in the intense degree of inflammation present.

In the initial stages the pathologic changes in the limb may give rise to serious apprehension, both for the integrity of the limb and the life of the patient.

It was taught by Cornil that in this phlegmasia, inflammatory changes first appeared in the lymphatics; while Epstein, and others of the German school, looked to the nerves and the vasa-vasorum, to their influence on the middle tunic. Here they found these infinitesimal feeders in places closed, dilated, or sacculated.

In progressive cases we will encounter every degree of tissue change, malnutrition in the limb, atrophy, or persistent œdema, or hyperplasia.

As in aggravated cases of simple varix, it will be noted that after fair functional restoration is established, the full vitality of the limb is diminished; impairment of power and mobility remaining throughout life.

Phlegmasia-dolens, it is fair to regard as one of those diseases incidental especially to child-bearing women, which through diseased changes in the veins may lead to atrophic conditions and varix, presenting special features.

It may be well, before leaving this important topic, to touch on the necessity of ascertaining, if possible, a rational view of the pathology of phlegmasia-dolens, in order to apply such knowledge to practicable purposes.

It may be well at the outset, to state my own convictions, after having examined and treated, in all, about twenty cases of milk-leg of various types and in various stages of the disease.

First. Notwithstanding the discordant views of pathologists on the morbid changes present, whether as a primary, intercurrent or ultimate condition, the veinous system of the affected limb is invariably involved; stasis and œdema appear *not* over the entire limb, but in the plexus of the vena-saphena, of the knee, of the deep femoral, in the gluteal. The patient complains of a sense of *weight* and *stiffness* in the limb, before pain and rigors set in.

This is the stage for aggressive relief measures, local and general, for posture, bandage-pressure, free leeching, capping, bathing, mercurial inunction, or other effective antiphlogistics. Internally, mercurial purgation, diaphoretics, or remedies of *specific* energy.

Second. Turgescence passing into inflammation, we have multiple phlebitis, thrombosis and extensive plastic exudate into all the soft parts, which, if of short duration, may be checked at its onset, and resolutive established.

Third. *All* the destructive effects of phlegmasia succeed upon advance and wide-spread effects of unchecked inflammation, want of relief to the circulation and the tissues, now in a state bordering on asphyxia, which, if unrestricted by art, proceeds to organic changes in the structures.

Fourth. The clinical history of phlegomasia-dolens, as well as its accentuated symptoms, point to a very close affinity to a malady of common occurrence, though practically, as little understood as itself, to *rheumatism*. Like rheumatism it evidently depends on some disordered state of the blood, with a predisposition to pathologic changes in the veins of one limb, the whole aggravated by the hæmic changes of pregnancy, the gravid uterus, the violent strain of delivery, and, perchance, exposure, or premature exercise after confinement.

There is no reason why we should not give the germs the "go-by" in this instance. To speak of "infection" is begging the question, as stagnant overheated clotted blood will *decompose* and decomposition without the microzyme as Bechamp long since pointed out is impossible.

Fifth. That the essence of this phlegmasia is quite identical with rheumatism is borne out by the *therapeutic test*, in the free local application of salicylic acid externally, and, the internal administration of the alkalies colchicum and sulphur will often demonstrate.

TRAUMATIC PHLEBITIS OR VARIX.

One of the first questions which we must ask ourselves in this connection, is—does phlebitis commonly follow or ensue in consequence of injuries to a limb? To which we may answer, unqualifiedly, yes!

But, how about varicose veins?

From *direct violence*, probably never; from over-strain of the limb, especially the use of the bicycle, commonly.

The older authors seemed to possess a singular fear of injury of the veins. Astley Cooper had witnessed the most dreadful consequences following ligatures of them. Guthrie, Larrie and other renowned military surgeons strongly inveighed against the practice.

Sometimes, however, we often ligate a vein with as little hesitation as an artery, and no harmful results.

In fractures, there is no doubt, but severe injury of the deep veins, is always followed by acute phlebitis; and it may be in some cases, the persistence of the phlebitis tends to aggravate pain, interfere with osseous union, and greatly retard restoration of functions.

In most complete fractures we have evidence of free escape of blood into the soft parts; some, from the ends of the fragments, but, no doubt more from the laceration of the deep veins. But resorption is usually rapid and if there has been numerous thrombi, they soon break down and disappear.

In the treatment of some thousands of fractures, in no instance has a case of sudden death occurred after injury imputable to displaced thrombi and pulmonary infarction.

Of late, since the practice of litigation in every instance of injury, that offers any possible pretext for damages, has become so general it appears that many are coming forward with "broken veins" which they allege were produced by falls, blows, abrasions, etc.

It would be well to secure the consensus of opinion of surgeons on this point.

In many thousand cases of traumatism which has come under my care, I have never known of *one* case of varicose veins of the limbs succeeding a fall or a blow. Never, after the very worst description of compound fracture, or after dislocation—the very worst possible injuries of a limb, short of its complete demolition.

*Hydraulic pressure* is a factor of the first importance, in the evolution of varix of a member, *physological*, *pathological*, *inci-*

*dental* or *extrinsic*, the latter only can be here briefly considered. By the act of expiration, the elevation of the diaphragm, and the cardiac vacuum in ventral diastole the venous current moves onward toward the cava. In various situations, in order to support the column of blood, to prevent a back-flow, or a great strain on the vessels, in sudden concussion of shock, the valves are provided. In saphenous varix we have evidence that valvular imperfection, is the initial step in pathologic changes; distention of the vessel, atrophy, rupture of the middle coat, phlebitis and thrombosis, following consecutively.

Various occupations entailing long standing and certain violent forms of athletic exercise, institute a type of trauma most prolific in the development of varix; particularly when a predisposition exists.

Sometimes there is no description of exercise so prolific in producing varicose lesions in the veins of the lower extremities, as the *awkward* or *immoderate* use of the bicycle. Pushing against a grade, the heart and the whole vascular system is put on a severe strain, while simultaneously with the violent efforts of the lower limbs, the alternate, rapid and extreme bending of the femoral vein, with each revolution of the wheel, the thigh is sharply flexed against the Poupart's ligament, while the body is inclined well forward.

Through this state of forced respiration and impediment to the return circulation, a great strain is put on the veins, their valves are ruptured, the venous walls over-distended or sundered, when phlebitis is established or excessive varix formed.

I am acquainted with no mode of exercise so prone to violence of the veins in the extremities and degenerative changes in them, as excessive bicycling; and what may be moderate with one may be beyond safe limits in another.

### PATHOLOGIC STATES INDUCED BY DISPLACEMENT OF THE COAGULATED, ORGANIZED, OR DISORGANIZED VENOUS THROMBI.

It is now quite generally acknowledged that endocarditic vegetation and emboli, carried from the heart in the arterial current are a most fertile source of occlusion of the cerebral vessels —of grave lesions of the brain.

But, it is most remarkable that in this age of progressive investigation, little or nothing has been done in the way of studying these probably only too common affections of the lungs, dependent on infarcts carried from the lumina of diseased veins; especially veins of the lower extremity; or those lesions of the liver consecutive to thrombotic impaction and diseases of the hemorrhoidal plexus.

This aspect of varix opens up a vast field of study in a practically unexplored territory, which it is hoped will soon engage the attention of an ambitious and experienced investigator. An elucidation of the subject on scientific lines will unquestionably tend to shed a flood of light on many now obscure points in the internal pathology of organs.

